# Knowledge, attitudes and behaviours towards vitamin D and sun exposure of parents of infants and young children and health professionals in New Zealand

**DOI:** 10.1177/02601060231185190

**Published:** 2023-07-02

**Authors:** Pamela von Hurst, Hajar Mazahery, Esme Reynolds, Alexandra Thomson, Mia Franklin, Cathryn Conlon

**Affiliations:** 1 College of Health, Massey University, Auckland, New Zealand

**Keywords:** Vitamin D, sun exposure, knowledge, attitudes, behaviours, parents, infants, children, health professionals, New Zealand

## Abstract

**Background:**

Vitamin D deficiency may result in adverse long-term health consequences in adulthood if it occurs during fetal development, infancy and childhood. To effectively improve vitamin D status of infants/toddlers, there needs to be knowledge and awareness of vitamin D among parents and health professionals.

**Aims:**

The aim of this study was to investigate parents’ and health professionals’ knowledge, attitudes and behaviours towards vitamin D and sun exposure over two timepoints.

**Methods:**

The study was an ecological study over two timepoints (Parents 2009 and 2021; Health professionals 2010 and 2019) and used an online questionnaire.

**Results:**

The analysis included 9834 parents (2009 n = 8032; 2021 n = 1802) and 283 health professionals (2010 n = 193; 2019 n = 90). Parents and health professionals had good knowledge of vitamin D sources, roles and risk factors for deficiency over two timepoints. There were however some confusions regarding the vitamin D content of breast milk, exclusive breastfeeding as a risk factor for deficiency, and ineffectiveness of sun exposure through glass windows in relation to vitamin D synthesis. In 2019, only 37% of health professionals indicated giving advice on supplements for infants/toddlers. Most parents and health professionals believed there was not enough information available to parents regarding vitamin D (>90%) and that skin cancer prevention messages make it difficult to get information about vitamin D across (>70%).

**Conclusion:**

Although parents and health professionals had good knowledge in most areas, knowledge of some specific sources and risk factors for vitamin D deficiency was poor.

## Introduction

Low vitamin D status in early life has important health implications for the developing fetus and mother, infants and young children and may result in adverse long-term health consequences in adulthood (Lucas et al., 2008). It has been well established that vitamin D has both musculoskeletal (Pasco et al., 2008) and extra-musculoskeletal health benefits in children (Urashima et al., 2010; Saggese et al., 2015; Mazahery et al., 2019). Furthermore, evidence suggests a possible association between maternal vitamin D deficiency and several complications of pregnancy including pre-eclampsia, gestational diabetes mellitus, pre-term birth and birthing an infant who is small for gestational age (Palacios et al., 2019).

Most vitamin D is obtained from endogenous synthesis within the skin after exposure to ultraviolet β (UVβ) radiation (Nowson et al., 2012). However, excessive exposure to ultraviolet radiation (UVR) is the main cause of skin cancers, putting New Zealanders at an increased risk due to high solar UVR levels (Wright et al., 2007; Gage et al., 2018). Consequently, New Zealand (NZ) has one of the highest rates of skin cancers globally (Whiteman et al., 2016). This is yet more concerning for infants due to their immature skin which puts them at greater risk of skin cancer (Shafie Pour et al., 2015; Ministry of Health, 2013a). Therefore, the promotion of sun safe messages has taken precedence over information on vitamin D (Ministry of Health, 2013a). In 2012, the Ministry of Health released a Consensus Statement on Vitamin D and Sun Exposure in NZ, followed by a Companion Statement for Pregnancy and Infancy in 2013 (Ministry of Health, 2012, 2013a), which was revised in 2020. The current advice is to only consider vitamin D supplementation for women during pregnancy and to breastfed infants who are ‘at risk’ of deficiency (Ministry of Health, 2020a). Risk factors for vitamin D deficiency include residing at higher latitudes, winter/spring season, complete sun avoidance, naturally darker skin colour, non-European ethnicity, having liver or kidney disease or taking certain medications, and specifically in infants and young children, breastfeeding practices, born pre-term or to vitamin D deficient mothers, or having a sibling with rickets (Ekeroma et al., 2015; Cairncross et al., 2016; Camargo et al., 2010; Delshad et al., 2019; Grant et al., 2009; Houghton et al., 2010; Wall et al., 2013; Rigo and Senterre, 2006).

Rickets, a consequence of severe vitamin D deficiency, continues to be identified amongst infants and children (Creo et al., 2017; Wheeler et al., 2015), and vitamin D deficiency (25(OH)D < 50 nmol/L) during pregnancy/lactation and infancy appears to be common in NZ (Camargo et al., 2010; Wall et al., 2013). A recent longitudinal study found a high prevalence of vitamin D deficiency (at least at one or two timepoints of assessment) among infants (76%) and their mothers (65%) (Wheeler et al., 2018). Vitamin D supplementation (only 28% were on supplement) was associated with higher 25(OH)D concentrations. Infants included in the above-mentioned studies were exclusively breastfed and commonly un-supplemented. Exclusive breastfeeding and not using vitamin D supplement is a risk factor for vitamin D deficiency. Unfortunately, there is no information available on vitamin D supplement use specific to NZ lactating mothers and infants at risk of vitamin D deficiency. As evidenced by the high prevalence of vitamin D deficiency among infants, NZ vitamin D policy appears to have issues in relation to its understandability, implementation or effectiveness (Wheeler et al., 2018).

In order to effectively improve vitamin D status of mothers and infants/toddlers, there needs to be increased knowledge and awareness of the importance of vitamin D and current health policies among parents and health professionals. Research has shown that increased knowledge among female adolescents is associated with intention to take vitamin D supplement (Alami et al., 2019), and increased parental awareness about recommendations is associated with intention to give vitamin D supplements to children (O’Dea et al., 2018). It should be noted that intention does not necessarily lead to action and core to the success of a health education programme is to include the factors that affect consumers’ supplementation intention in education or marketing programmes (e.g. attitudes, subjective norms). As health professionals are an essential element of the social support network of parents during their child's early stages of life, they have an important role in delivering information about vitamin D and sun exposure guidelines to parents (Rossiter et al., 2019). Furthermore, health professionals have a key role in identifying those who are at risk of deficiency and devising suitable management plans (Dix et al., 2017). To the best of authors’ knowledge, there is currently no or limited information available on parents’ and health professionals’ level of knowledge on vitamin D in NZ. Therefore, the current study aimed to identify gaps in parents’ and health professionals’ knowledge in relation to vitamin D in NZ, and to compare the knowledge, attitudes and behaviours before and after the release of the Ministry of Health statements.

## Methods and materials

This online ecological study consisted of two surveys: parents’ and health professionals’ surveys. The parents’ survey collected data in 2009 and 2021 and health professionals’ survey in 2010 and 2019. Participation in 2021 and 2019 were not likely to include individuals that participated in the 2009 and 2010 surveys, respectively.

The study was deemed a low-risk research project by the Massey University Human Ethics Committee. Consent was obtained from all participants.

### 2009 and 2021 Parents’ surveys

**Survey populations:** Participants in the 2009-parent survey were recruited through ReachMe (a marketing website popular with parents in NZ at the time), and the 2021-parent survey through Facebook groups for parents of young children across NZ. Participants were included in these surveys if they had a child 5 years of age or younger, lived in NZ at the time of survey and were able to understand written English.

**Survey questionnaire (Supplemental material 1):** The online questionnaire used in 2021 was re-administered from 2009. This allowed for a comparison to be made over the two timepoints. The original questionnaire was developed following a comprehensive review of the literature and identification of guidelines, and a round table consultation with experts in the nutrition field. Once completed, an expert panel reviewed the questionnaire, and it was tested in a sample of mothers in NZ to ensure it was suitable for use within this population group. Following the development of the questionnaire in 2009, new guidelines and recommendations on vitamin D and sun exposure were published in NZ by the Ministry of Health. To address this, three questions were added in the 2021 questionnaire, and these include:

- Have you seen the Ministry of Health resources on vitamin D and sun exposure for pregnancy and your baby?
- Do you know the current recommendations for vitamin D and sun exposure?
- Do you know there is a funded vitamin D supplement available for babies at risk of vitamin D deficiency?

Additional answer responses were also included for some questions in the 2021 questionnaire, for example, when assessing participants’ sources of information, social media was added as an option due to the increase in popularity of this platform over the past decade.

The updated questionnaire consisted of 51 questions, categorised into five sections; general vitamin D knowledge, knowledge within the NZ context, attitudes towards vitamin D and sun exposure and typical behaviours related to pregnancy and youngest child, followed by participants’ characteristics. Most questions were single answer and forced response and a small number included ‘other’ option with an open text box asking participants to provide further information. Knowledge questions were primarily statements with multiple choice answers (e.g. good, moderate, poor or unsure, true, false or unsure) and some asked participants to list answers. Attitude questions were statements with Likert scale answer options (strongly agree, agree, neither agree or disagree, disagree, and strongly disagree), and typical behaviours were Likert scale questions with always, usually, sometimes, rarely, or never options.

### 2010 and 2019 Health professionals survey

**Survey populations:** Health professionals were recruited by sending invitations to several relevant organisations (Dietitians NZ, NZ Nurses Organisation, Plunket and NZ College of Midwives), to health professionals listed in Massey University's contact database, and local medical centres. Also, the survey was posted in closed Facebooks of organisations which gave permission (Dietitians NZ and NZ Nurses Organisation). Participants were included if they indicated employment and/or hold an approved qualification required to practice as a health professional. Health professions included in this study were limited to those recognised by the Health Practitioners Competence Assurance Act as these professions are not permitted to practice outside of their scope (Ministry of Health, 2020b). Participants with incomplete survey response were excluded from this survey. No further inclusion or exclusion criteria were defined.

**Survey questionnaire (Supplemental material 2):** The online questionnaire used in 2019 was re-administered from 2010. This allowed for a comparison to be made over the two time points. The 2010 survey was developed based on three documents: Cancer Society of NZ's Position Statement on the Risks and Benefits of Sun Exposure in NZ (Cancer Society of New Zealand, 2007), Food and Nutrition Guidelines for both Healthy Infants and Toddlers (Ministry of Health, 2008), and Healthy Pregnant and Breastfeeding Women (Ministry of Health, 2006). The survey was not updated with the release of the 2012 NZ Consensus Statement on Vitamin D.

The survey consisted of 41 questions, categorised into five sections; general vitamin D knowledge, knowledge within the NZ context, awareness of current recommendations, current practices regarding management of vitamin D status, followed by participants’ characteristics. Questions were forced response and mostly giving the option to select unsure/do not know.

## Statistical analyses

Statistical analyses were performed using IBM SPSS version 27.0 (IBM Corp; Armonk, NY, USA). A two-sided P < 0.05 was considered statistically significant. Descriptive statistics, numbers and proportions, were used to describe the variables. The change in parents’ and health professionals’ knowledge, and attitudes over time (from 2009 to 2021 for parents and from 2010 to 2021 for health professionals) was investigated using cross-tabulation. Participants not completing all five sections of the survey were excluded from the analyses.

## Results

### Participant characteristics

**Parents*:*** In total, 9834 parents were included in the final analysis (2009 survey: n = 8032 and 2021 survey: n = 1802, Figure 1). Initially, 9220 and 2418 participants attempted the 2009 and 2021 survey, respectively, of whom 1188 (not having a child <5 years old and duplicate attempts) and 616 (not consenting to participate, not having a child <5 years old, incomplete survey) were excluded.

**Figure 1.**
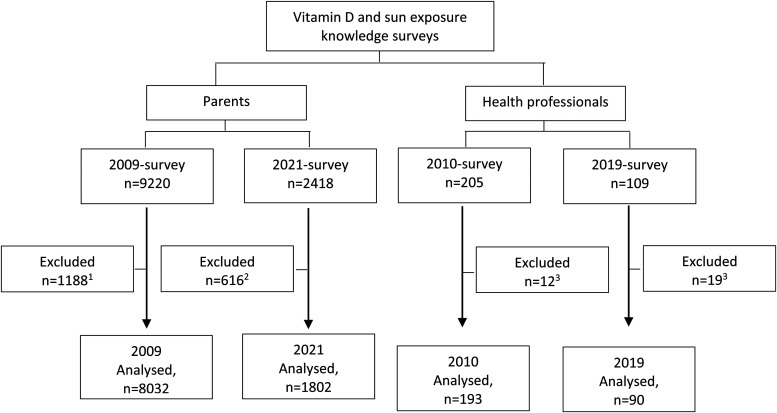
Study flow diagram. ^1^Reasons for exclusion: not having a child under 5 years of age at the time of completing survey or completing the questionnaire multiple times. ^2^Reasons for exclusion: not consenting to participate, youngest child being over the age of 5 years, or not completing all five sections of the survey. ^3^ Reasons for exclusion: not meeting the occupation criteria or not stating occupation.

Compared to the 2009 survey, a larger proportion of parents in 2021 were ≥35 years old (42 vs. 32%, P < 0.001), having a child 3 to 5 years old as their youngest child (24 vs. 14%, P < 0.001), and having tertiary level education (78 vs. 59%, P < 0.001), but smaller proportion were NZ European (70 vs. 75%, P < 0.001, Table 1).

**Table 1.** Demographic characteristics of parents and health professionals.

**Demographic characteristics of parents**			
	**2009 Survey**	**2021 Survey**	P-value*
Age of mothers, n (%)			
< 25 years	869 (11)	87 (5)	<0.001
25 to <30 years	1775 (22)	294 (16)	
30 to <35 years	2762 (35)	664 (37)	
≥35 years	2576 (32)	750 (42)	
Age of youngest child, n (%)			
<1 years	3386 (42)	542 (30)	<0.001
1 to 3 years	3488 (44)	826 (46)	
3 to 5 years	1130 (14)	433 (24)	
Ethnicity, n (%)			
NZ European	5984 (75)	1256 (70)	<0.001
Māori	812 (10)	209 (12)	
Pacific	216 (3)	38 (2)	
Other	992 (12)	299 (16)	
Highest level of education, n (%)			
Secondary	2170 (27)	249 (14)	<0.001
University/professional	4706 (59)	1410 (78)	
Trade or technical	868 (11)	128 (7)	
Other	239 (3)	6 (0.3)	
**Demographic characteristics of health professionals**	**2010 Survey**	**2019 Survey**	P-value*
Gender, n (%)			
Female	185 (96)	88 (99)	0.18
Male	8 (4)	1 (1)	
Ethnicity, n (%)			
NZ European	148 (83)	67 (75)	<0.001^1^
Māori	21 (12)	7 (8)	
Pacific	6 (3)	0 (0)	
Other	4 (2)	15 (17)	
Occupation, n (%)			
Dietitian/nutritionist	28 (15)	26 (29)	<0.001^2^
Midwife	8 (4)	22 (24)	
General Medical Practitioner	15 (8)	2 (2)	
Nurse (including Plunket nurse)	133 (69)	33 (37)	
Other	8 (4)	7 (8)	
Years of experience, median (25th, 75th percentiles)	15 (5, 28)	8 (4, 17)	<0.001

Abbreviations: NZ = New Zealand.

*Chi-square test for categorical variables and Mann-Whitney U test for not normally distributed continues variable.

^1^ Two cells had expected counts <5.

^2^ One cell had expected count <5.

**Health Professionals:** In total, 283 health professionals were included in the final analysis (2010 survey: n = 193 and 2019 survey: n = 90, Figure 1). Initially, 205 and 102 participants attempted the 2010 and 2019 survey, respectively, of whom 19 and 12 were excluded (not meeting occupation inclusion criteria).

In both surveys, most health professionals were women and of NZ European ethnicity (75% in 2010 vs. 83% in 2019, P < 0.001, Table 1). Compared to the 2010 survey, a larger proportion of health professionals in 2019 were nutritionist/dietitians (29 vs. 15%) or midwives (24 vs. 4%,), but smaller proportion were nurses (37 vs. 69%) or general medical practitioners (2 vs. 8%) (P < 0.001). Median (25th and 75th percentiles) years of experience decreased between the two populations, 15 (5, 28) versus 8 (4, 17) years in 2010 and 2019, respectively (P < 0.001).

### Knowledge of sources and roles of vitamin D and risk factors for vitamin D deficiency

**Parents*:*** More than 60% of parents in both surveys correctly identified the role of vitamin D in bone health (63% in 2009 vs. 61% in 2021, P = 0.12, Table 2). However, a larger proportion of parents in 2021 were aware of its role in immunity than 2009 (48 vs. 29%, P < 0.001). Most parents in both surveys correctly identified sunshine as the single most important source of vitamin D (89 vs. 95% in 2009 and 2021, respectively). Although the proportion of parents who correctly identified poor sources increased from 2009 to 2021 (P < 0.001), only 12% identified breast milk and 44% cow's milk as poor sources in 2021.

**Table 2.** Parent's and health professional's knowledge about roles and sources of vitamin D and risk factors for vitamin D deficiency.

**Parents**			
Questions **	**2009 Survey**	**2021 Survey**	P-value*
Role of vitamin D, n (%)			
Bone health	5059 (63)	1104 (61)	0.12
Immune system	2332 (29)	868 (48)	<0.001
Incorrect answer/do not know	3312 (41)	800 (44)	0.02
Single most important source of vitamin D, n (%)			
Sunshine (skin synthesis)	7115 (89)	1694 (95)	<0.001
Fortified foods/supplements	167 (2)	22 (1)	
Natural foods	706 (9)	74 (4)	
Correctly identified good and poor dietary sources of vitamin D for infants and toddlers, n (%)			
Good sources			
Infant formula	1800 (23)	600 (34)	<0.001
Toddler formula	1613 (20)	410 (23)	0.01
Poor sources			
Breast milk	311 (4)	217 (12)	<0.001
Cow's milk	1956 (25)	777 (44)	<0.001
Correctly ranked high, moderate and low risk factors for vitamin D deficiency for infants and toddlers^1^, n (%)			
High risk factors			
Born to vitamin D deficient mothers	3373 (42)	1277 (71)	<0.001
Being exclusively breastfed	159 (2)	88 (5)	<0.001
Having darker skin	559 (7)	400 (22)	<0.001
Not regularly exposed to sunlight	4616 (58)	1289 (72)	<0.001
Moderate risk factors			
Living in South Island	1293 (16)	904 (50)	<0.001
Extensive use of sunscreen	2174 (27)	798 (44)	<0.001
Low risk factor			
Being formula fed	3278 (41)	1100 (61)	<0.001
**Health professionals**	**2010 Survey**	**2019 Survey**	**P-value**
Role of vitamin D, n (%)			
Calcium absorption	120 (82)	81 (90)	0.08
Bone health	114 (78)	85 (94)	<0.001
Immune system	50 (34)	65 (72)	<0.001
Incorrect answer	36 (25)	24 (27)	0.71
Single most important source of vitamin D, n (%)			
Sunshine (skin synthesis)	138 (95)	85 (95)	0.61
Fortified foods/supplements	2 (1)	0 (0)	
Natural foods	4 (3)	4 (4)	
Dietary sources of vitamin D for infants and toddlers, n (%)			
Correctly identified good sources			
Infant or toddler formula	98 (70)	48 (53)	0.01
Fortified cow's milk	75 (53)	55 (61)	0.24
Incorrectly identified the following foods as good sources			
Breast milk	66 (47)	28 (31)	0.02
Unfortified cow's milk	28 (20)	19 (21)	0.82
Correctly identified high risk factors for vitamin D deficiency for infants and toddlers, n (%)			
Born to vitamin D deficient mothers	121 (63)	63 (71)	0.22
Being exclusively breastfed	27 (14)	29 (33)	<0.001
Having darker skin	100 (52)	75 (84)	<0.001
Covering skin	134 (70)	77 (87)	<0.01
Not regularly exposed to sunlight	165 (86)	87 (98)	<0.01
Correctly identified high risk factors for vitamin D deficiency for pregnant and lactating women, n (%)			
Having darker skin	92 (48)	79 (89)	<0.001
Being housebound	150 (79)	83 (93)	<0.01
Covering skin	166 (87)	83 (93)	0.12

*Chi-squared test; significant at P < 0.05.

** Multiple responses were allowed, unless otherwise stated.

Similarly, there was an increase in parents’ knowledge of risk factors for vitamin D deficiency in infants/toddlers over time (P < 0.001). In 2021, 72% correctly identified no regular sun exposure, 71% maternal vitamin D deficiency, 22% darker skin, and only 5% exclusive breastfeeding as high risk factors.

**Health Professionals:** More than 75% of health professionals in both surveys, with a larger proportion in 2019, correctly identified the role of vitamin D in calcium absorption (82% in 2010 vs. 90% in 2019, P = 0.08, Table 2) and bone health (78% in 2010 vs. 94% in 2019, P < 0.001). Similarly, a larger proportion of health professionals in 2019 were aware of its role in immunity than 2009 (72 vs. 34%, P < 0.001). Most health professionals in both surveys correctly identified sunshine as the single most important source of vitamin D (95% in both surveys). There was however some confusion around dietary sources of vitamin D for infants/toddlers with 31% and 21% in 2019 incorrectly identified breast milk and cow's milk, respectively, as good sources. Health professionals’ knowledge about breast milk improved over time (P = 0.02), but about cow's milk remained at the same level (P = 0.82).

Health professionals’ knowledge of risk factors for vitamin D deficiency in infants/toddlers improved over time (P < 0.001) with most identifying no regular sun exposure (98%), covering skin (87%), dark skin (84%) and maternal vitamin D deficiency (71%) as risk factors in 2019. However, despite seeing an improvement in 2019, knowledge of breast milk was still poor with only 33% identifying it as a risk factor. Only 9% (n = 17) and 25% (n = 22) of health professionals identified all four high risk factors in 2010 and 2019 (P < 0.001). Similarly, health professionals’ knowledge of risk factors for vitamin D deficiency during pregnancy/lactation improved over time with approximately 90% correctly identifying dark skin, being homebound and covering skin as risk factors in 2019. Half (n = 80) and 82% (n = 73) of health professionals identified all three risk factors in 2010 and 2019, respectively (P < 0.001).

### Knowledge of sun exposure and awareness of guidelines

**Parents:** Although parents’ knowledge of the impact of season, sun exposure through windows, the amount of skin exposed and skin colour on vitamin D synthesis improved overtime (P < 0.001), approximately 30–50% did not correctly answer these questions in 2021 (Table 3). Also, more than 60% of parents in 2021 did not correctly identify current guidelines regarding sun exposure during summer for both infants/toddlers and pregnant/lactating women.

**Table 3.** Parent's and health professional's knowledge of sun exposure in relation to vitamin D synthesis and awareness of current guidelines.

	**Parents**			**Health professionals**		
	**2009 Survey**	**2021 Survey**	**P-value**	**2010 Survey**	**2019 Survey**	**P-value**
**Knowledge of sun exposure in relation to vitamin D synthesis**						
During winter vitamin D status may drop below adequate levels, n (%)						
Correct answer (true)	5636 (70)	1410 (78)	<0.001	157 (81)	82 (91)	0.04
Incorrect answer (false)/unsure	2368 (30)	392 (22)		36 (19)	8 (9)	
Amount of time needed in the sun to make enough vitamin D depends on the amount of skin exposed, n (%)						
Correct answer (true)	3198 (40)	948 (53)	<0.001	112 (58)	73 (81)	<0.001
Incorrect answer (false)/unsure	4805 (60)	854 (47)		81 (42)	17 (19)	
With dark skin need to spend longer in the sun to make enough vitamin D, n (%)						
Correct answer (true)	1913 (24)	772 (43)	<0.001	104 (54)	71 (79)	<0.001
Incorrect answer (false)/unsure	6091 (76)	1030 (57)		89 (46)	19 (21)	
Sun exposure through a window is just as effective as outdoor sun exposure in relation to vitamin D synthesis, n (%)						
Correct answer (false)	2618 (33)	712 (40)	<0.001	91 (47)	47 (52)	0.43
Incorrect answer (true)/unsure	5386 (67)	1090 (60)		102 (53)	43 (48)	
**Current guidelines for sun exposure for infants/toddlers**						
In summer, parents are recommended to expose baby's face and arms to 5 (for light skin) to 20 min (for dark skin) of direct sunlight per day before 11am and after 4pm^1^, n (%)						
Correct answer (true)		630 (35)		111 (58)	41 (47)	0.05
Incorrect answer (false)/unsure		1172 (65)		81 (42)	49 (53)	
During winter and spring, infant and toddlers should spend time outside in the sun to maintain adequate vitamin D levels^1^, n (%)						
Correct answer (true)		1497 (83)		166 (86)	80 (89)	0.50
Incorrect answer (false)/unsure		305 (17)		27 (14)	10 (11)	
**Current guidelines for sun exposure for pregnant/lactating women**						
Between October and March pregnant and lactating women are recommended to expose their face and arms to 5 to 20 min of sunshine per day^1^, n (%)						
Correct answer (true)		609 (34)		107 (55)	47 (52)	0.61
Incorrect answer (false)/unsure		1193 (66)		86 (45)	43 (48)	
Most pregnant women will get enough vitamin D in summer through incidental sun exposure outside peak UV times, n (%)						
Correct answer (true)	3036 (38)	789 (44)	<0.001	103 (53)	50 (56)	0.73
Incorrect answer (false)/unsure	4968 (62)	1013 (56)		90 (47)	40 (44)	
Deliberate sun exposure during the middle of the day is recommended for pregnant/lactating women, n (%)						
Correct answer (false)	4472 (56)	757 (42)	<0.001	146 (76)	66 (73)	0.68
Incorrect answer (true)/unsure	3532 (44)	1045 (58)		47 (24)	24 (27)	

Abbreviations: NZ = New Zealand; UV = ultraviolet.

^1^ These statements were not included in parents’ 2009 survey.

**Health Professionals:** Health professionals’ knowledge of the impact of season, amount of skin exposed and skin colour on vitamin D synthesis improved overtime (P < 0.05) with 80% or more identifying correct answers in 2019 (Table 3). However, there was some confusion around the impact of glass on vitamin D synthesis with approximately 50% of health professionals in both surveys (P = 0.43) thought that sun exposure through window is as effective as outdoor sun exposure in relation to vitamin D synthesis. There was no improvement in health professionals’ awareness of current guidelines in relation to sun exposure for both infants/toddlers and pregnant/lactating women with approximately 50% in both surveys incorrectly identified guidelines for sun exposure during summer.

### Parents’ awareness of current recommendations/policies for vitamin D and sun exposure

The majority of parents reported not being aware of current recommendations for vitamin D and sun exposure for their baby/child (87%) and not seeing the Ministry of Health resources on vitamin D (93%). No further analyses were performed (unbalanced sample size) to investigate the effect of awareness on parents’ knowledge.

### Parents’ attitudes towards sun exposure

In both surveys, 90% of parents agreed that children need some direct sunlight to be healthy (Table 4). However, most parents thought sunbathing was harmful (87% in 2009 vs. 78% in 2021, P < 0.001) and were worried about detrimental effect of direct sunlight on child's skin (81% in 2009 vs. 80% in 2021, P = 0.22) and the link between direct sunlight to skin cancer (86% in 2009 vs. 83% in 2021, P < 0.001).

**Table 4.** Parents’ attitude towards vitamin D and sun exposure.

	**2009 Survey**	**2021 Survey**	P-value*
Children need to get some direct sunlight to be healthy, n (%)			
Agree	7225 (90)	1620 (90)	0.41
Neutral	528 (7)	132 (7)	
Disagree	251 (3)	50 (3)	
If skin is always protected from the sun it can put people at risk of vitamin D deficiency, n (%)			
Agree	4380 (55)	1173 (65)	<0.001
Neutral	2526 (32)	433 (24)	
Disagree	1098 (13)	196 (11)	
Sunbathing is harmful to my child, n (%)			
Agree	6979 (87)	1396 (78)	<0.001
Neutral	684 (9)	272 (15)	
Disagree	341 (4)	134 (7)	
I worry that sun exposure is linked to skin cancer, n (%)			
Agree	6918 (86)	1501 (83)	<0.001
Neutral	833 (10)	212 (12)	
Disagree	253 (4)	89 (5)	
I worry that the sun will damage my child's skin, n (%)			
Agree	6469 (81)	1441 (80)	0.22
Neutral	1062 (13)	234 (13)	
Disagree	473 (6)	126 (7)	

*Chi-squared test; significant at P < 0.05.

### Health professionals’ views on the availability of information and current practices

In both surveys, a large proportion of health professionals indicated that there is insufficient information about vitamin D for parents (95% in 2010 vs. 94% in 2019, P = 0.77) and health professionals (81% in 2010 vs. 74% in 2019, P = 0.22). Furthermore, more than 70% of health professionals in both surveys (79% in 2010 vs. 73% in 2019, P < 0.001) indicated that skin cancer prevention messages make it difficult to get messages about vitamin D across. However, in both surveys, 59% indicated giving advice on increasing sun exposure if concerned about vitamin D deficiency in infants/toddlers (an increase from 49% in 2010, P = 0.11) or pregnant/lactating women (an increase from 50% in 2010, P = 0.18).

Health professionals’ awareness of vitamin D supplements for infants and toddlers increased over time (42% in 2010 vs. 56% in 2019, P = 0.03). However, only 9% in 2010 and 37% in 2019 indicated giving advice on taking vitamin D supplement if concerned about vitamin D deficiency in infants/toddlers.

In both surveys, approximately 40% of health professionals indicated that the multiple vitamin and mineral supplements designed for women to take during pregnancy contained vitamin D, of whom approximately 20% did not know if pregnant women can meet their requirements for vitamin D from supplements only. Health professionals’ confidence in giving advice on vitamin D supplements for pregnant/lactating women improved over time (19% in 2010 vs. 53% in 2019, P < 0.001).

## Discussion

This is the first study in NZ to investigate both parents’ and health professionals’ knowledge and attitudes towards vitamin D and sun exposure over two timepoints (12 and 9 years apart, respectively). The results indicate that the level of knowledge of vitamin D in most areas was good and improved in both population groups over time. The increase in vitamin D knowledge however cannot be solely attributed to the increased awareness of population as the participants’ characteristics were different at two timepoints. Compared to the earlier surveys, a larger proportion of parents in the latest survey had higher educational level (78% vs. 59%) and were older (79% vs. 67% were >30 years old). Also, a larger proportion of health professionals were dietitians/nutritionists (29% vs. 15%) and midwives (24% vs. 4%). Age, educational level and specialisation have been shown to be indicators of vitamin D knowledge level (Sim et al., 2010; Christides, 2018; Zgliczyński et al., 2021; Al-Agha et al., 2016; Alemu and Varnam, 2012).

In the latest surveys, more than 90% of both parents and health professionals correctly identified sun exposure as the most important source of vitamin D, a finding consistent with that of other studies among parents from the United Kingdom, Ireland, Jordan, and Saudi Arabia (Aitken and Mushtaq, 2015; Al-Qudah et al., 2021; Alshamsan and Bin-Abbas, 2016; Drury et al., 2015; Toher et al., 2014) and among health professionals from NZ (Reeder et al., 2013). Despite an improvement, parents’ and health professionals’ specific knowledge of dietary food sources for infants/toddlers (e.g., infant/toddler formula as a good source and cow's milk and breast milk as poor sources) remained poor. A large proportion of parents (96% in 2009 and 88% in 2021) and approximately one in three of health professionals (47% in 2010 and 31% in 2019) were unaware that breast milk is a poor source of vitamin D for infants/toddlers. Breast milk is the preferred feeding method for infants and has nutritional, immunological and psychological benefits for both the infant and mother, however it is not a good source of vitamin D. However, it should be noted that, and as previously discussed, breast milk can be an adequate source if mother is supplemented with vitamin D or mother has regular sun exposure. Breastfeeding rate in NZ is high, with 97% of mothers initiating breastfeeding and 66% continuing to breastfeed at 6 months (Castro et al., 2017), reflecting the strong promotion of exclusive breastfeeding for the first 6 months of life in NZ.

Furthermore, despite the availability of NZ public health policy regarding vitamin D supplementation for infants (Ministry of Health and Cancer Society of New Zealand, 2012; Ministry of Health, 2008; Cancer Society of New Zealand, 2007), only a small proportion of parents and health professionals selected supplement as a source of vitamin D. Supplementation guidelines specify that only infants at risk of deficiency may require vitamin D supplementation. Although 78% of health professionals in 2019 were aware of current policies in NZ, and more than half were aware of supplements available for infants/toddlers, a large proportion (approximately 70%) indicated not giving advice on supplements for infants/toddlers. Not giving advice on vitamin D supplementation could be partly explained by health professionals’ misconception of the vitamin D content of breast milk (Oberhelman et al., 2018; Sherman and Svec, 2009), and not being aware of all risk factors for vitamin D deficiency in infants/toddlers. Only 25% of health professionals identified all risk factors, meaning that some risk factors may be overlooked in infants/toddlers. These findings coupled with the high rate of breastfeeding in NZ suggest that more education is required to increase health professionals’ awareness of breast milk vitamin D content, all risk factors for vitamin D deficiency and vitamin D supplementation policies for infants/toddlers to ensure the right information is delivered to parents and vitamin D deficiency is prevented and managed in a timely manner in at risk infants/toddlers.

Interestingly, health professionals were more confident in giving advice on vitamin D supplementation for pregnant/lactating women. Vitamin D supplementation in large doses has been shown to safely increase the vitamin D content of breast milk, providing adequate concentrations to support infants’ nutritional requirements (Dawodu and Tsang, 2012; Hollis et al., 2015; Wagner et al., 2006). However, the efficacy of such large dose supplementation has not been validated in larger and more representative populations of lactating women. Accordingly, recommendations to universally supplement breastfeeding mothers with a large dose of vitamin D is not advised. Therefore, relatively low-dose supplementation is only recommended for at risk women during pregnancy and lactation in NZ.

Both parents’ (from 7% to 22%) and health professionals’ (from 52% to 84%) awareness of darker skin as a risk factor for vitamin D deficiency has improved over the intervening decade, with parents having an incomparably poorer awareness than health professionals. This lack of awareness among parents is consistent with the findings of a study among Malaysian parents (Abu-Hassan et al., 2018). Darker skin contains higher concentrations of the pigment melanin, which interferes with vitamin D synthesis in the skin by absorbing UVβ radiation (Webb et al., 2018; D'Orazio et al., 2013). In NZ, preschool children and infants/toddlers with darker skin have been shown to have lower vitamin D status than those with lighter skin (Camargo et al., 2010; Grant et al., 2009; Cairncross et al., 2017). The lack of knowledge in this area is concerning as the population in NZ is becoming increasingly diverse with regards to skin colour and ethnicity (Callister et al., 2011). Thus, improvements in knowledge of this risk factor are required to decrease the prevalence of vitamin D deficiency in at risk populations.

Knowledge of sun exposure in relation to vitamin D synthesis for both population groups was mixed with some improvements seen in some areas over time. In the latest surveys, more than half of parents (approximately 50–78%) and most health professionals (approximately 80–90%) knew the impact of winter, skin colour, and the amount of skin exposed to sun on vitamin D synthesis. However, 60% of parents and 48% of health professionals did not know the impact of sun exposure through glass windows on vitamin D synthesis. Sun exposure outside of the peak sunlight hours is necessary as UVβ must come into contact with the skin directly to stimulate cutaneous vitamin D synthesis because UVβ cannot penetrate through glass windows. Exposure to sunlight through glass windows, however, is not safer because carcinogenic UVα is able to penetrate through glass (Khan et al., 2018). Therefore, this incorrect belief among both parents and health professionals may result in unnecessary sun exposure without the benefits of absorbing UVβ.

Most parents agreed upon the health benefits of direct sun exposure, nevertheless more than 70% were concerned about detrimental effect of sun exposure on their children's skin. Yet <50% of health professionals in 2019 (and even lower proportion in 2010, only 35%) correctly identified current guidelines regarding sun safety behaviours during summer despite the increase in relevant guidelines availability following 2010. With such an uncertainty, approximately 60% of health professionals indicated giving advice on increasing sun exposure if they were concerned about vitamin D deficiency in infants/toddlers or pregnant/lactating women. This is concerning as parents may receive incorrect advice on the use of sun protection measures from health professionals. New Zealand has one of the highest rates of skin cancer internationally, secondary to a comparably harsher UVR environment (McKenzie, 2017). Sun exposure in relation to vitamin D adequacy therefore, must not compromise on sun safety behaviours, particularly in the infant population whose skin is more susceptible to UVR-induced damage (Shafie Pour et al., 2015).

Strong public health campaigns focusing on cancer prevention are present to address the increased risk of skin cancer in NZ. More than 70% of parents and health professionals believed that skin cancer prevention messages make it difficult to get information about vitamin D across. Similar findings were also echoed in studies of parents in the United Kingdom (Littlewood and Greenfield, 2018) and health professionals in NZ and Australia (Bonevski et al., 2012; Reeder et al., 2012). The authors found it challenging to understand conflicting messages and achieve a balance between vitamin D requirements and the need for sun protection (Littlewood and Greenfield, 2018).

Both parents and health professionals agreed that there was not enough information available to parents about vitamin D. In 2013, fact sheets were published for parents and pregnant women by NZ Ministry of Health in several different languages that detailed sources of vitamin D, risk factors for deficiency and indicators for supplementation (Ministry of Health, 2013b, 2013c). It is possible that health professionals in 2019 were unaware of this resource or do not believe it is adequate. Furthermore, more than 70% believed that there was not enough information for health professionals. Other studies among health professionals in NZ and Australia also asked for clear guidelines in relation to the management of adequate vitamin D status (Bonevski et al., 2012; Reeder et al., 2012). These findings warrant further research into why current clinical guidelines/policies do not meet the needs of or are not appropriately communicated to health professionals.

These surveys had some limitations, including the exclusion of non-English reading participants, disproportionate participation within smaller ethnic groups, and uneven inclusion of health professions. Therefore, the results may not be representative of all the ethnic groups, non-English reading participants, and different health professions within NZ.

The current findings provide useful insight as to areas where parents’ education, and professional development of health professionals may be needed, thus potentially reducing the prevalence of vitamin D deficiency in the NZ population. The results of this study indicate that specific knowledge of dietary food sources (e.g. infant/toddler formula as good sources and cow's milk and breast milk as poor sources), risk factors for vitamin D deficiency in infants/toddlers (e.g. exclusive breastfeeding), and ineffectiveness of sun exposure through glass windows in relation to vitamin D synthesis was poor. Furthermore, health professionals were not confident in giving advice on vitamin D supplementation. These findings highlight the need for new strategies to communicate vitamin D health information to parents and for effective interventions to improve knowledge and confidence of health professionals.

## Supplemental Material

sj-docx-1-nah-10.1177_02601060231185190 - Supplemental material for Knowledge, attitudes and behaviours towards vitamin D and sun exposure of parents of infants and young children and health professionals in New Zealand Supplemental material, sj-docx-1-nah-10.1177_02601060231185190 for Knowledge, attitudes and behaviours towards vitamin D and sun exposure of parents of infants and young children and health professionals in New Zealand by Pamela von Hurst, Hajar Mazahery, Esme Reynolds, Alexandra Thomson, Mia Franklin and Cathryn Conlon in Nutrition and Health

sj-docx-2-nah-10.1177_02601060231185190 - Supplemental material for Knowledge, attitudes and behaviours towards vitamin D and sun exposure of parents of infants and young children and health professionals in New Zealand Supplemental material, sj-docx-2-nah-10.1177_02601060231185190 for Knowledge, attitudes and behaviours towards vitamin D and sun exposure of parents of infants and young children and health professionals in New Zealand by Pamela von Hurst, Hajar Mazahery, Esme Reynolds, Alexandra Thomson, Mia Franklin and Cathryn Conlon in Nutrition and Health

sj-docx-3-nah-10.1177_02601060231185190 - Supplemental material for Knowledge, attitudes and behaviours towards vitamin D and sun exposure of parents of infants and young children and health professionals in New Zealand Supplemental material, sj-docx-3-nah-10.1177_02601060231185190 for Knowledge, attitudes and behaviours towards vitamin D and sun exposure of parents of infants and young children and health professionals in New Zealand by Pamela von Hurst, Hajar Mazahery, Esme Reynolds, Alexandra Thomson, Mia Franklin and Cathryn Conlon in Nutrition and Health
