# Supplementary material for: Knowledge, attitudes and behaviours towards vitamin D and sun exposure of parents of infants and young children and health professionals in New Zealand
Source: Nutr Health. 2023 Jul 2;32(2):613–24. doi: 10.1177/02601060231185190 (PMC13096620; doi:10.1177/02601060231185190)

**Knowledge, attitudes and behaviours towards vitamin D and sun exposure of parents of infants and young children and health professionals in New Zealand**

**Supplementary material 2:** **Health professionals’ knowledge, attitudes and behaviours towards vitamin D and sun exposure questionnaire**


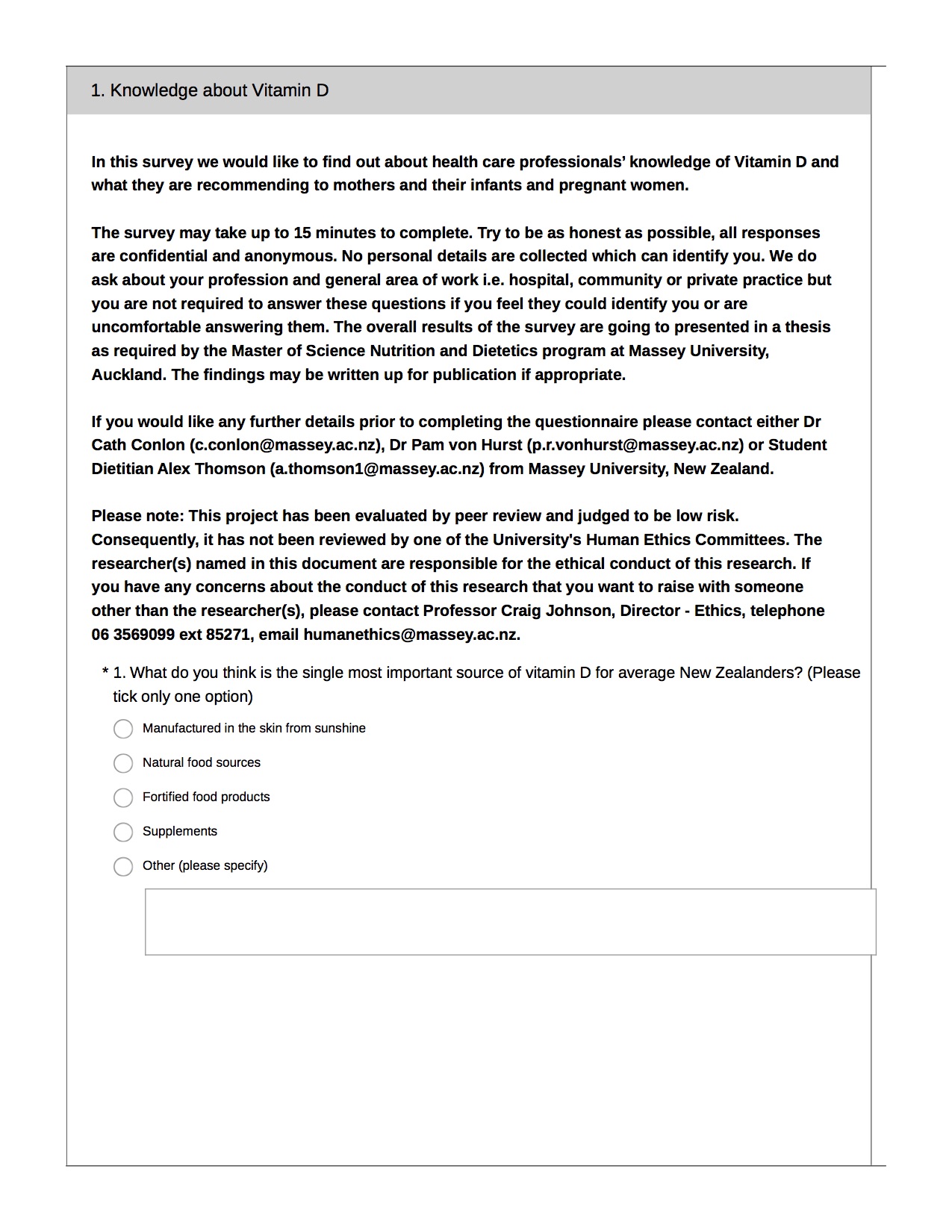


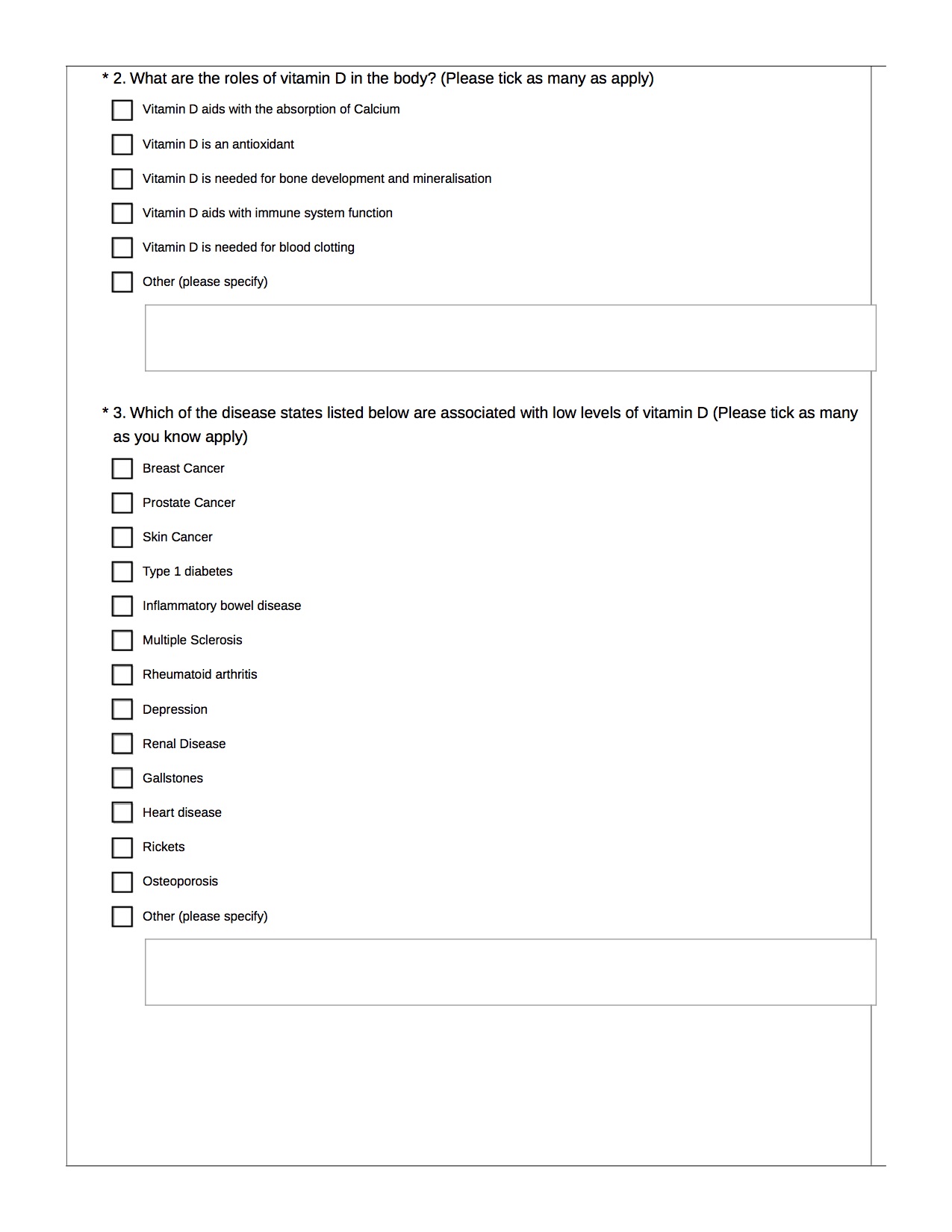


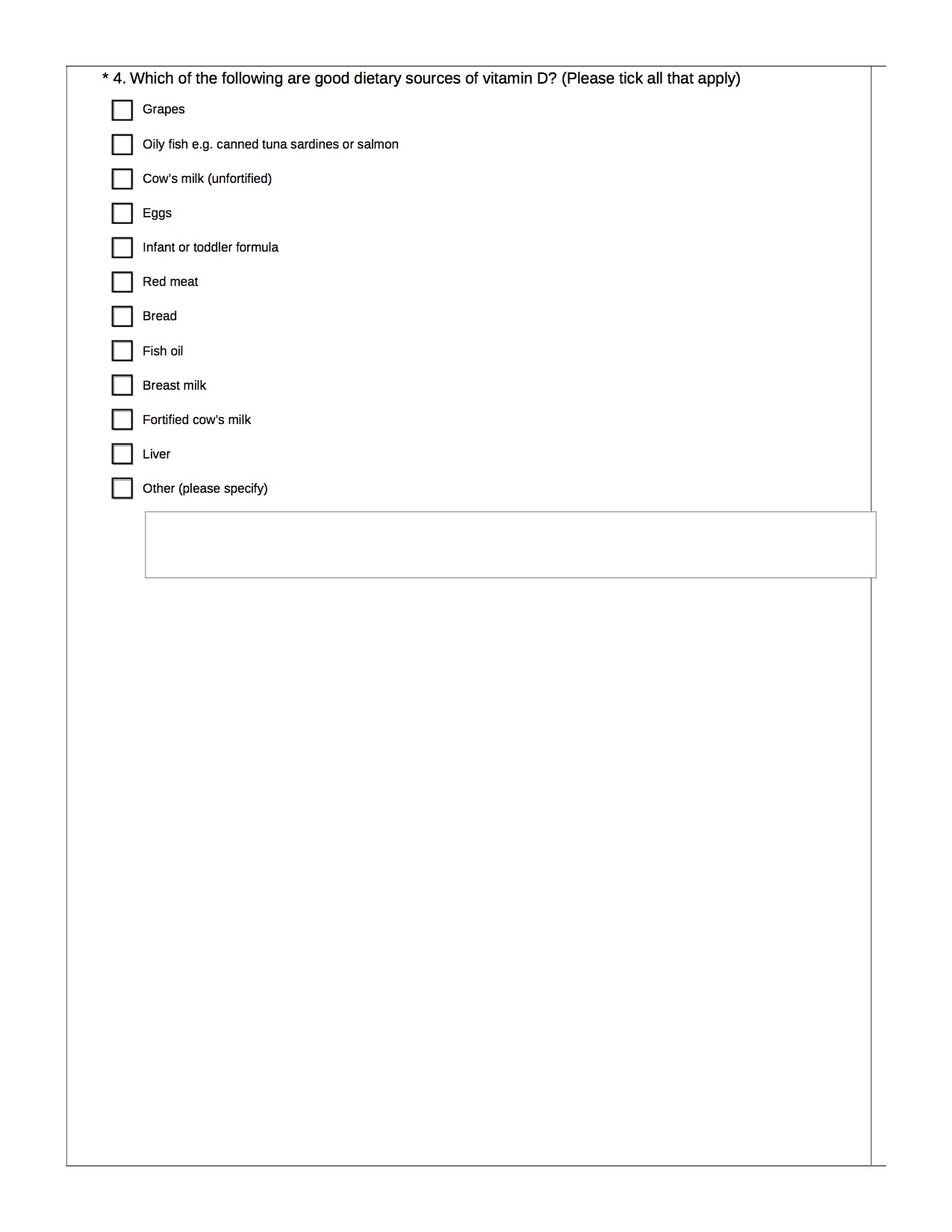


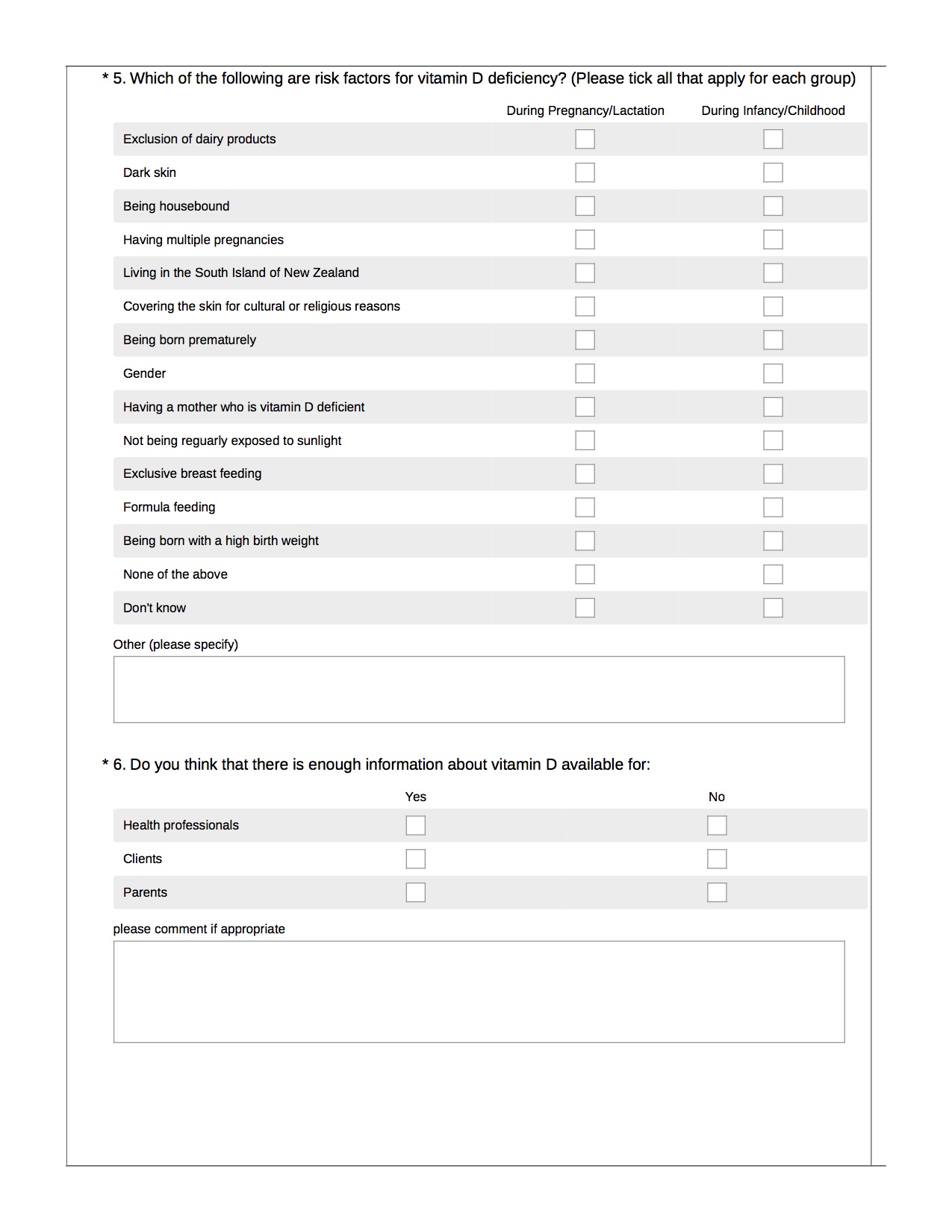


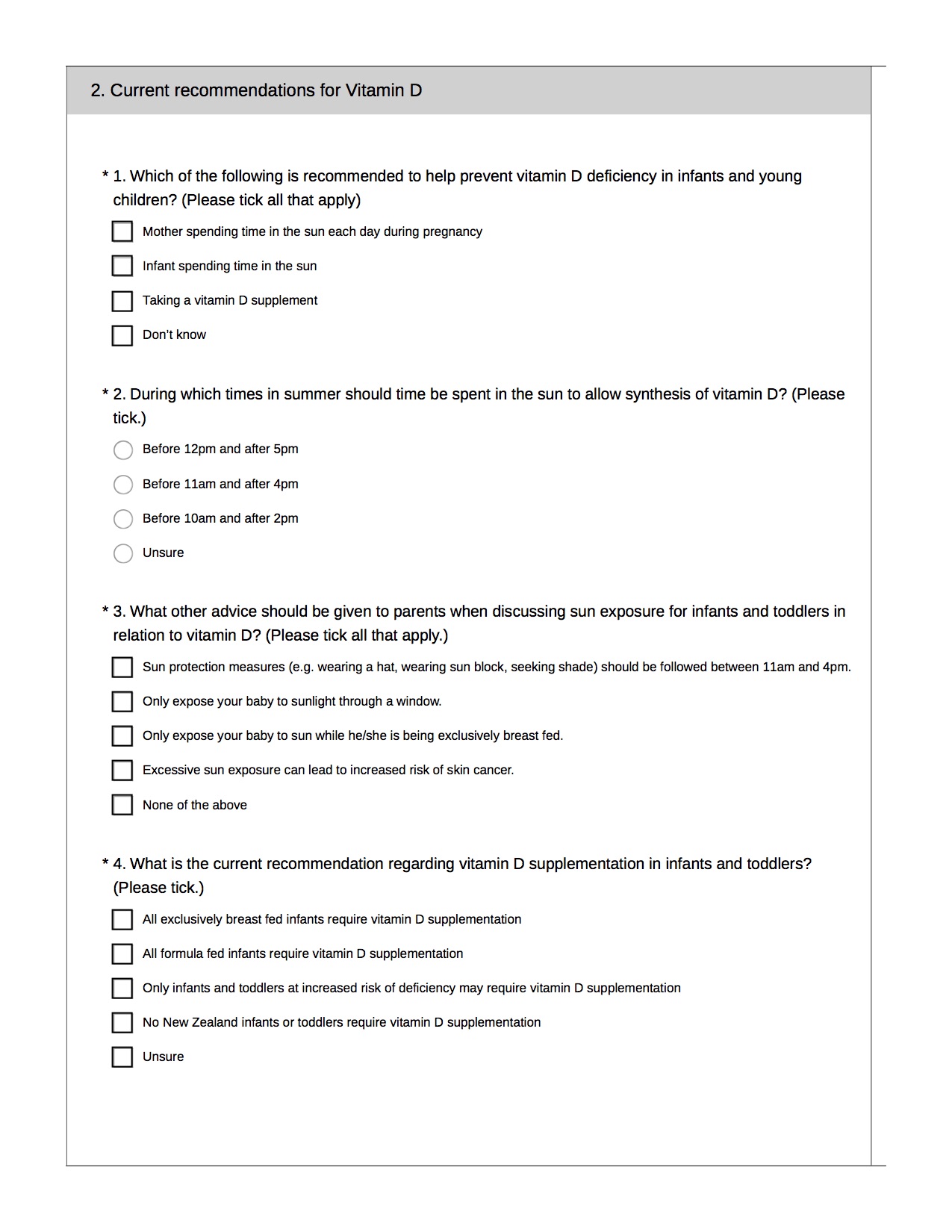


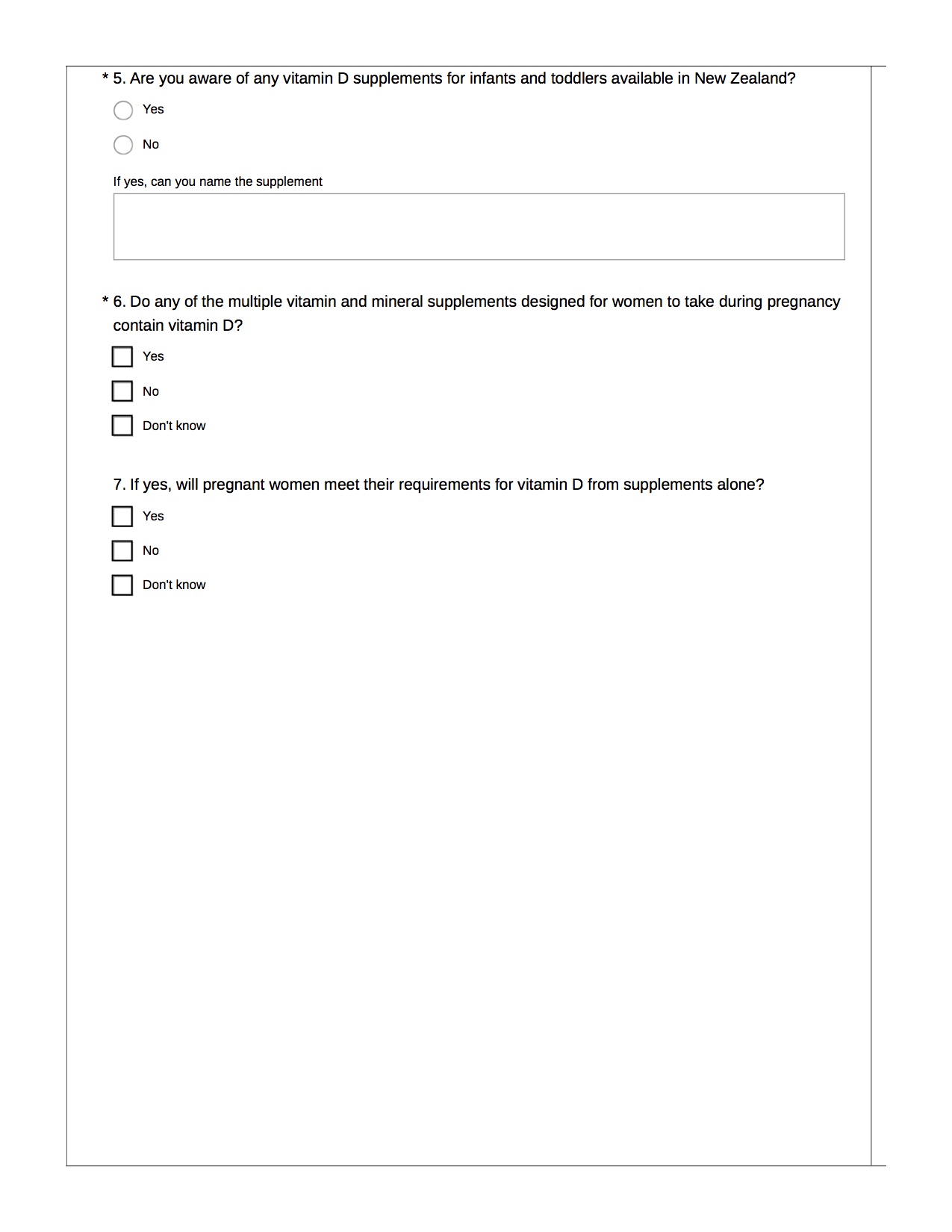


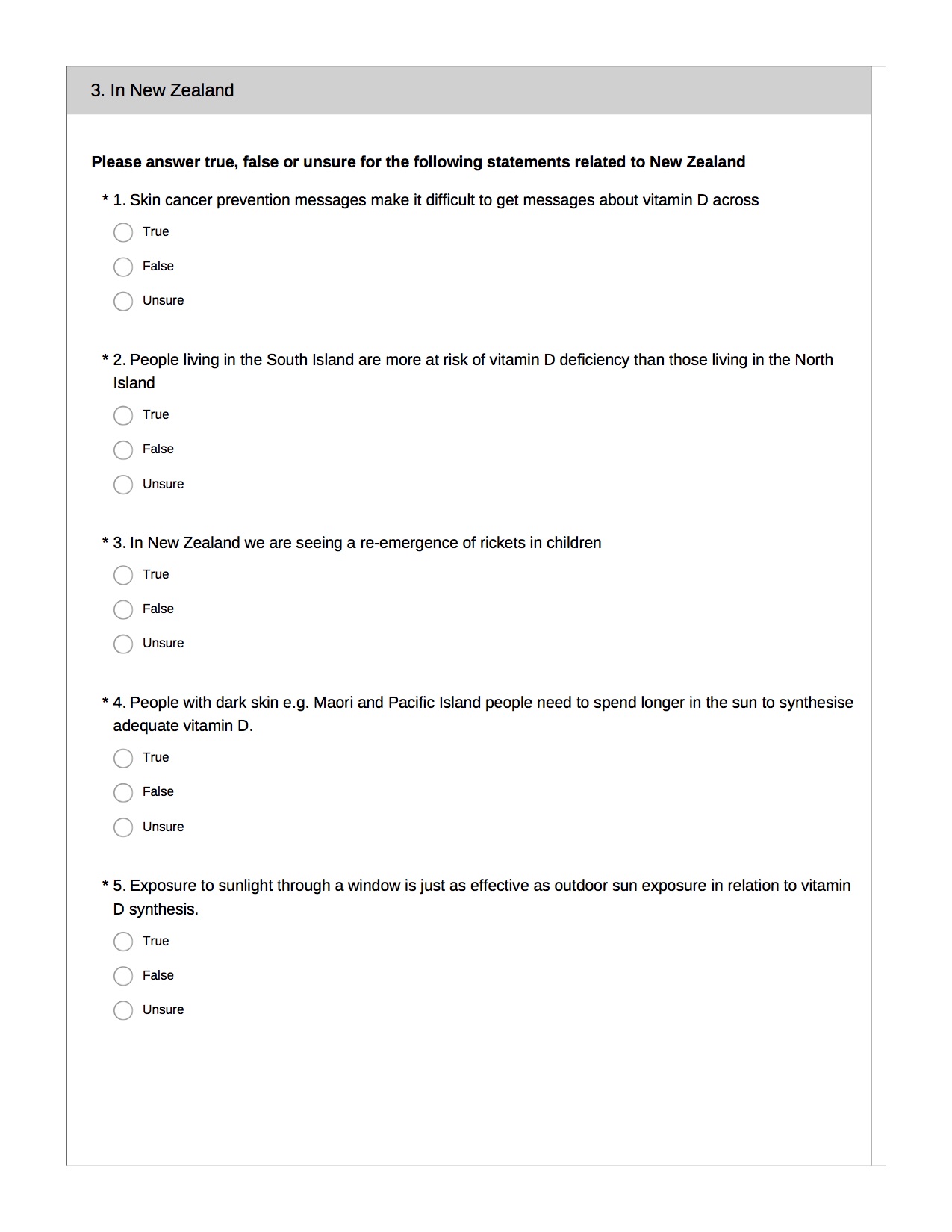


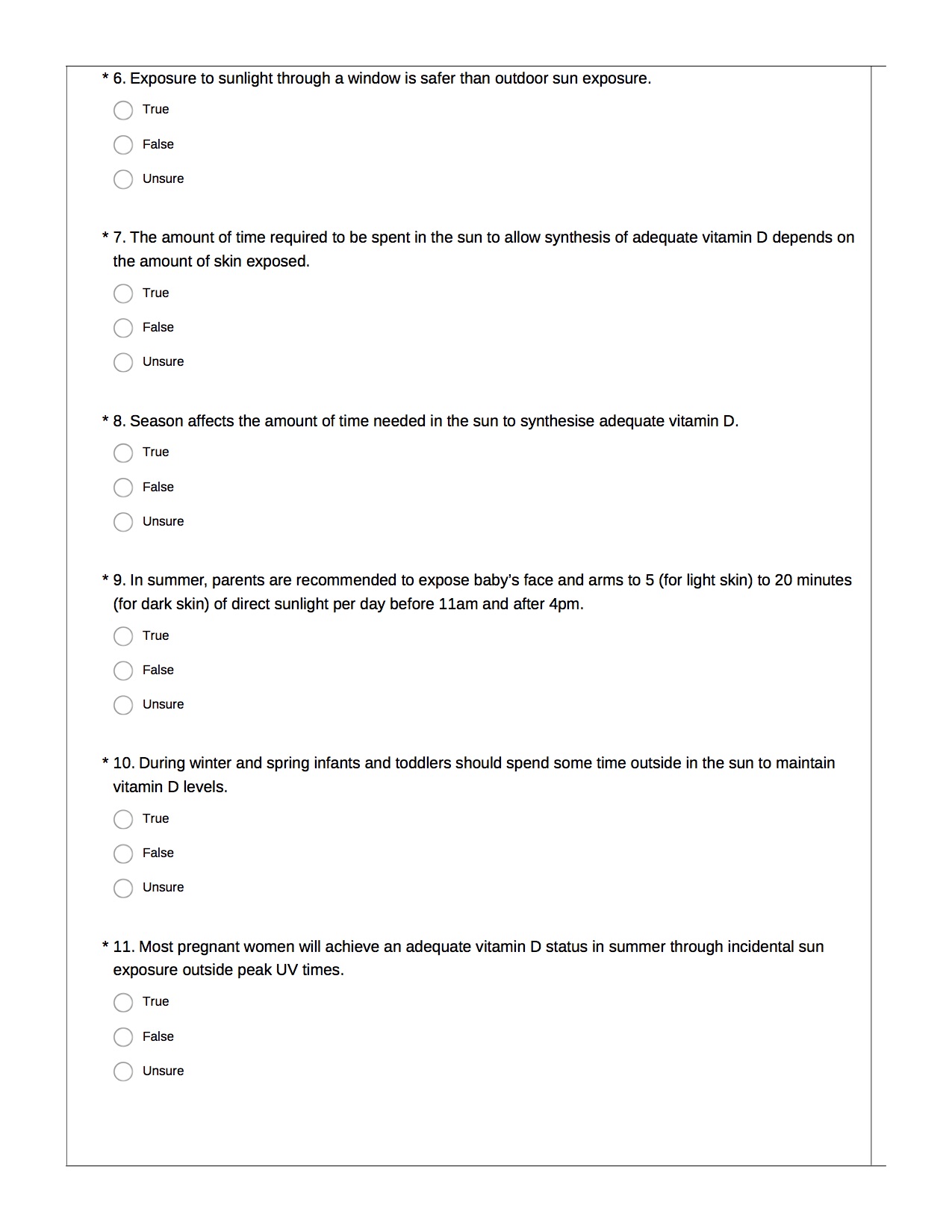


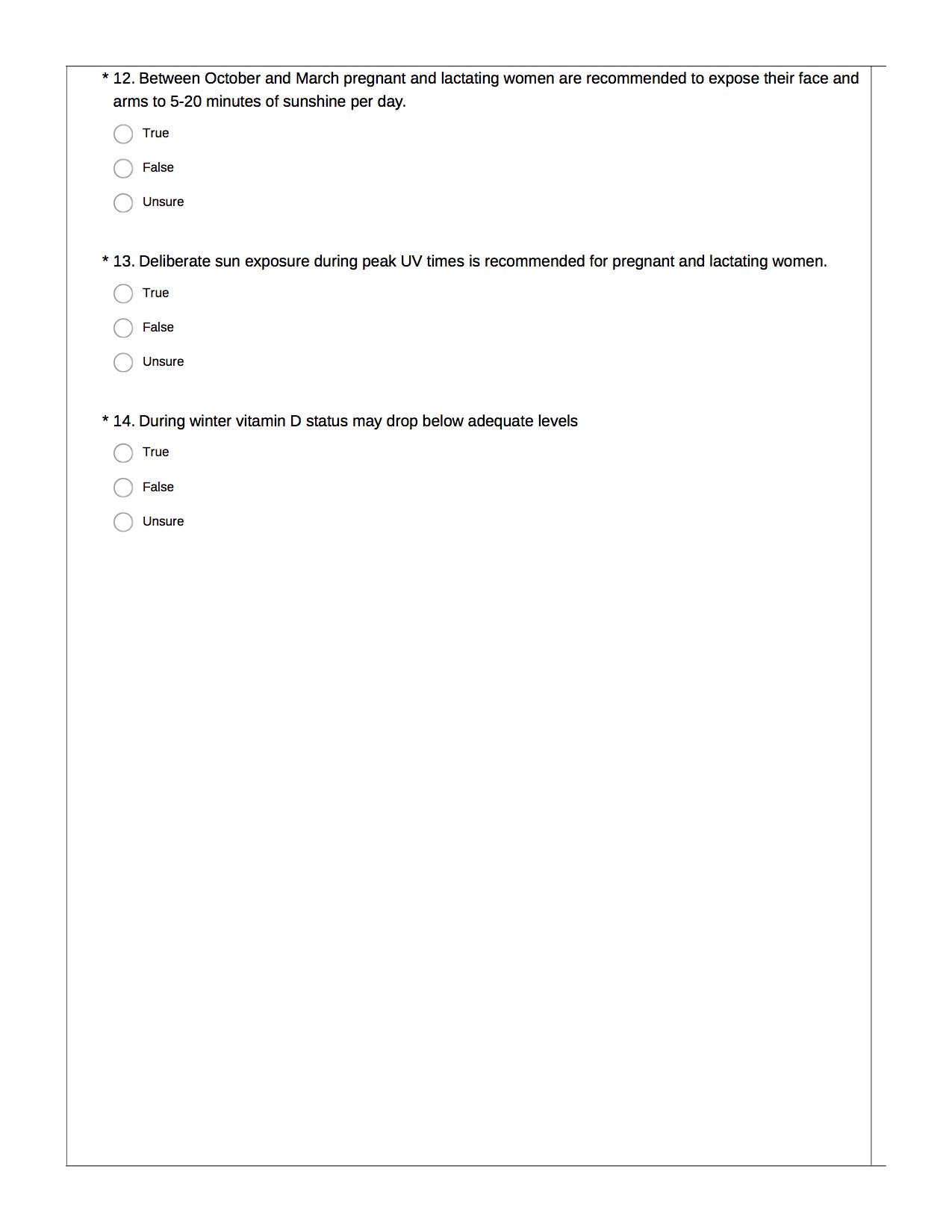


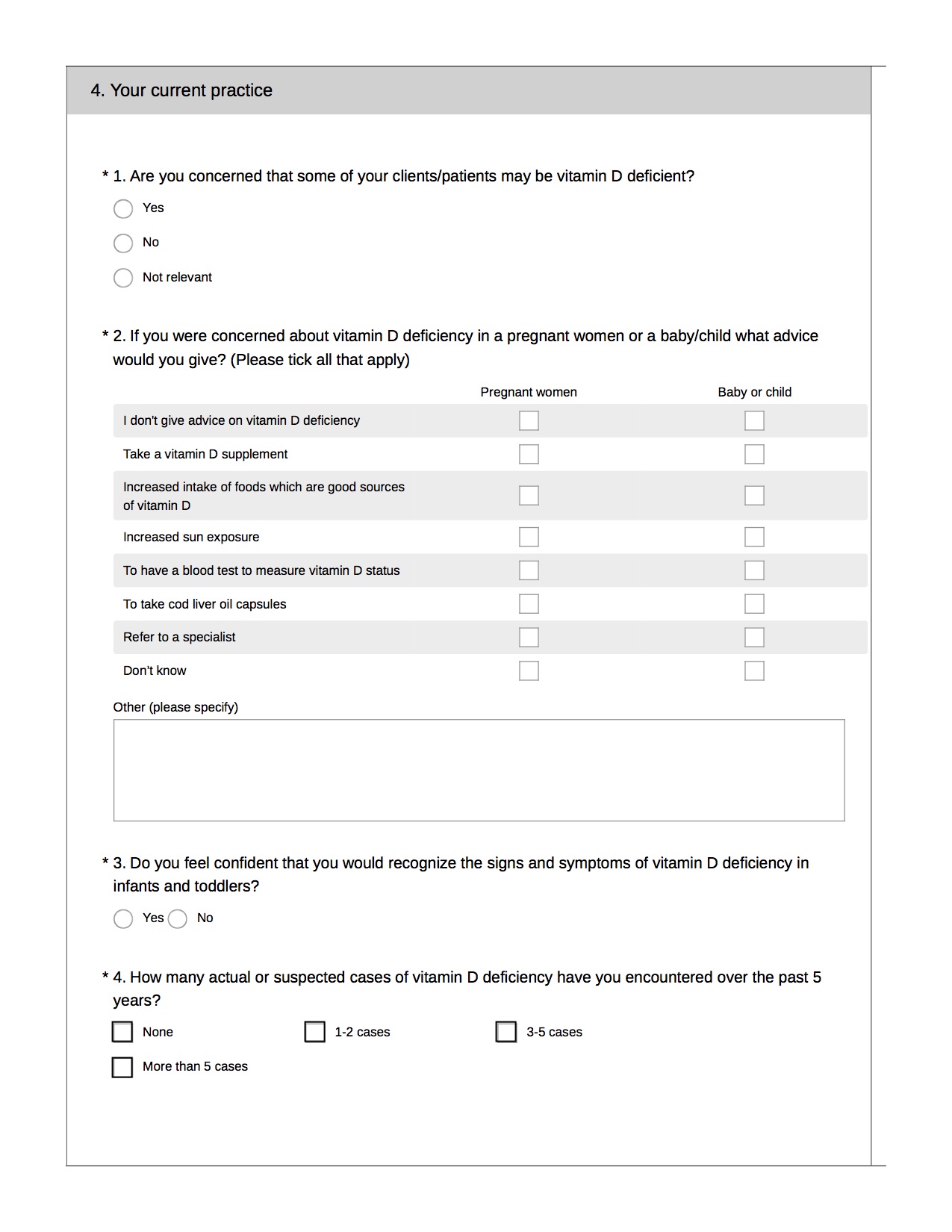


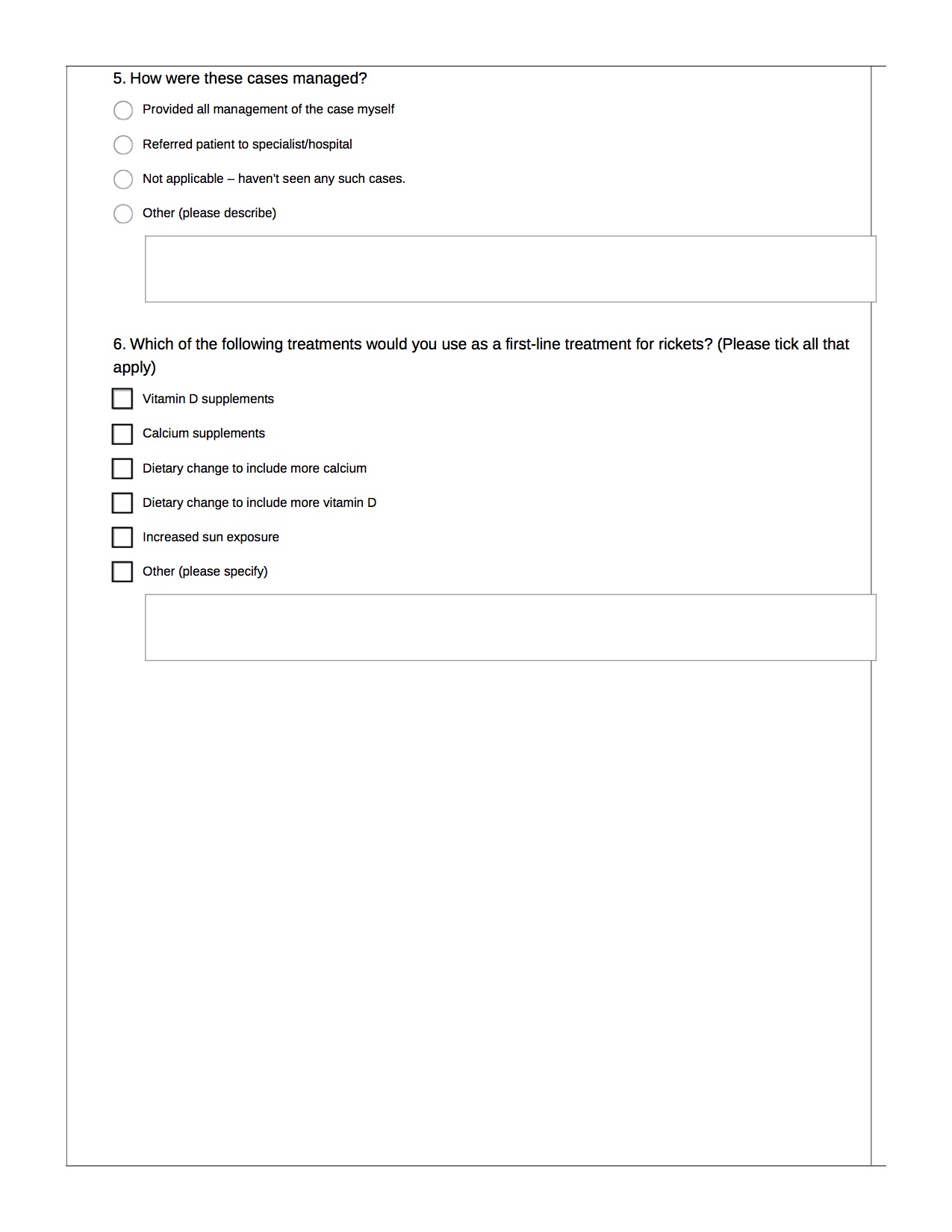


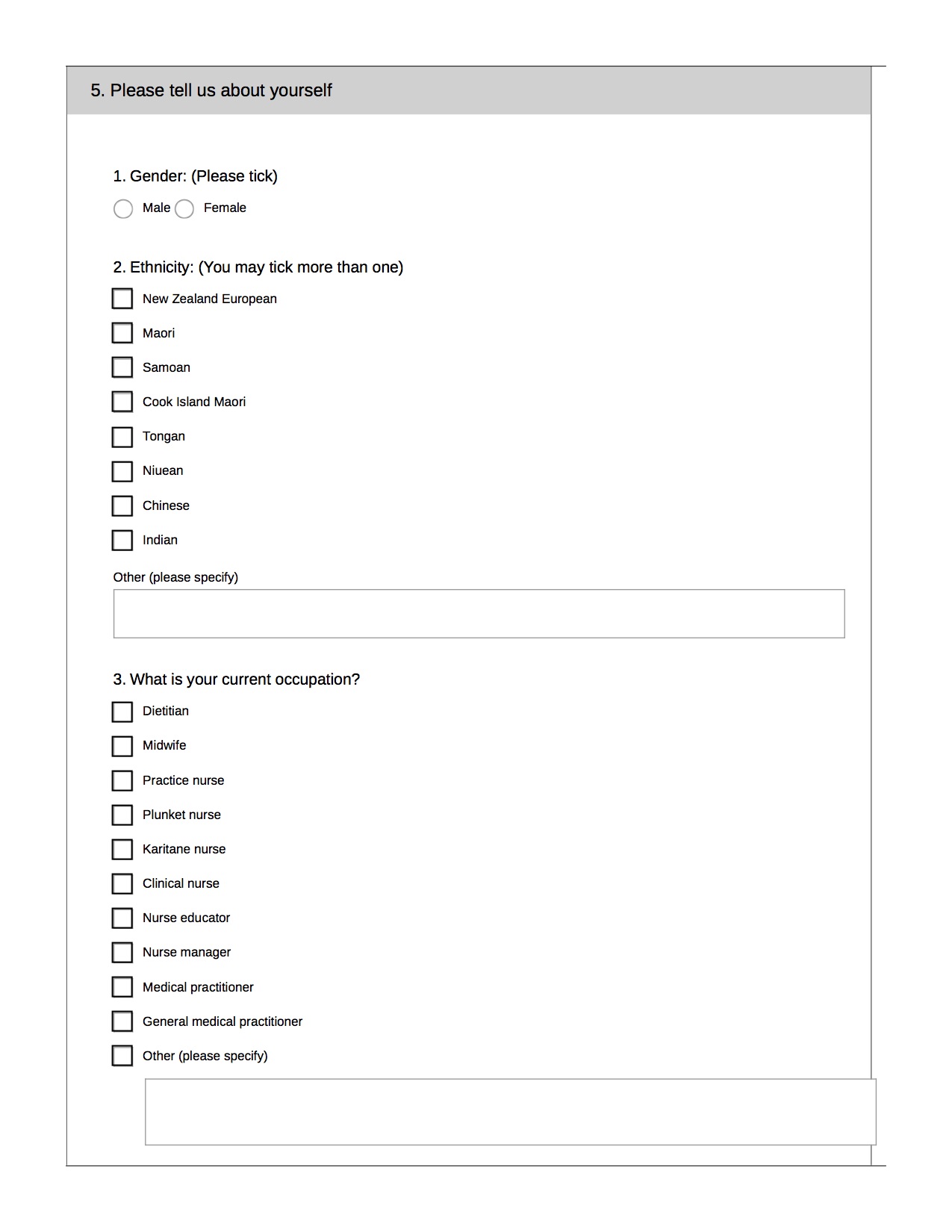


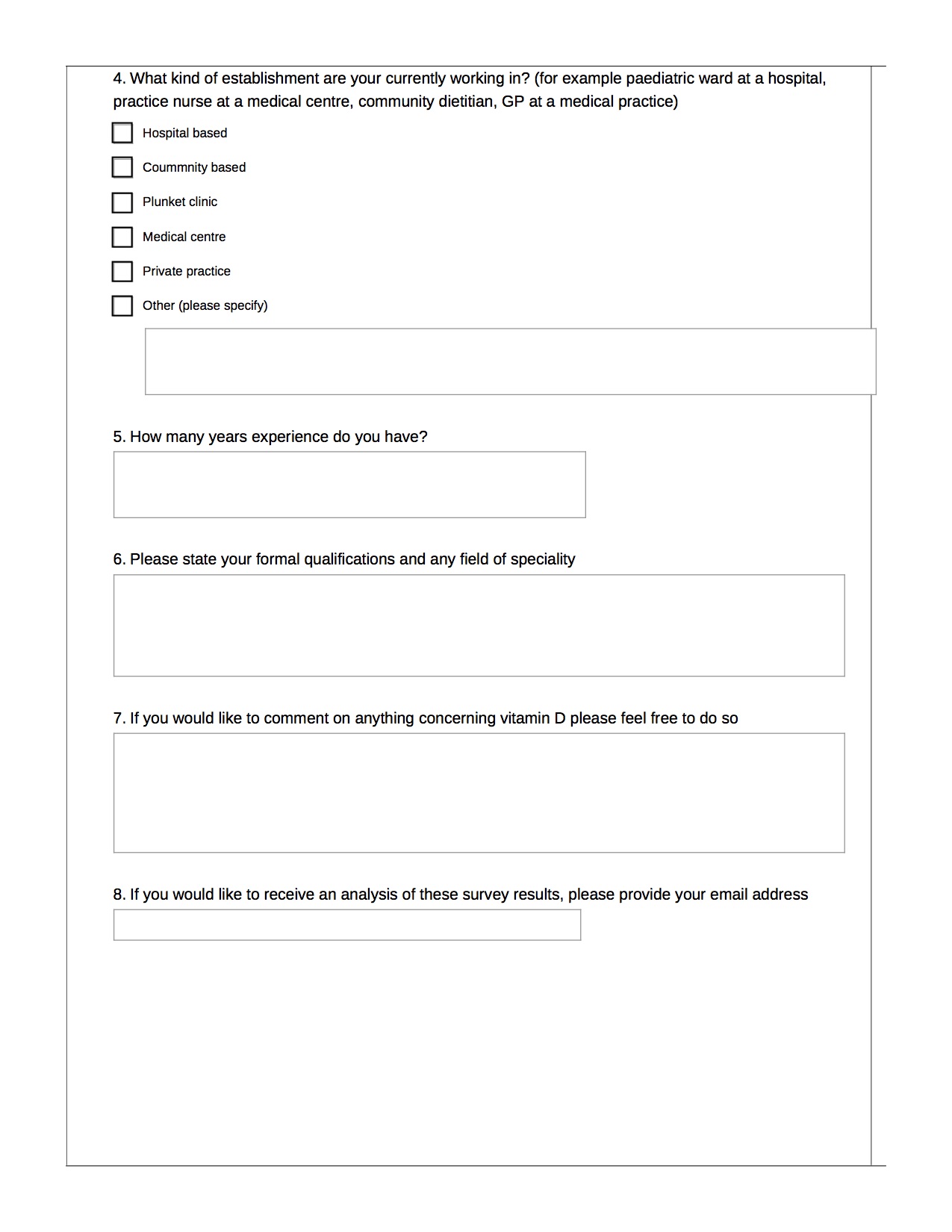

Supplement: sj-docx-2-nah-10.1177_02601060231185190 - Supplemental material for Knowledge, attitudes and behaviours towards vitamin D and sun exposure of parents of infants and young children and health professionals in New Zealand [file sj-docx-2-nah-10.1177_02601060231185190.docx]
